# 非小细胞肺癌免疫治疗进展

**DOI:** 10.3779/j.issn.1009-3419.2014.03.17

**Published:** 2014-03-20

**Authors:** 圆 何, 长宣 尤

**Affiliations:** 1 510515 广州，南方医科大学2013级研究生 Graduate Student of Southern Medical University, Guangzhou 510515, China; 2 510515 广州，南方医科大学南方医院肿瘤科 Department of Oncology, Nanfang Hospital, Southern Medical University, Guangzhou 510515, China

**Keywords:** 肺肿瘤, 免疫治疗, 进展, Lung neoplasms, Immunotherapies, Progress

## Abstract

肺癌是全球范围内癌性死亡的首要因素，发病率、死亡率高，预后较差，急需开发一种新的高效低毒疗法。作为术后辅助或是姑息治疗手段，免疫治疗为非小细胞肺癌患者提供了一个新的治疗方向。免疫疗法作用机理各不相同，如免疫检测点受体抑制剂（抗CTLA4抗体、抗PD-1抗体、抗PD-L1抗体）、主动性免疫疫苗（L-BLP25脂质体疫苗、Belagenpumatucel-L疫苗、MAGE-A3蛋白疫苗）、过继性免疫疫苗（CIK细胞）等，研究表明免疫治疗非小细胞肺癌肿瘤缓解率较前提高，前景值得期待，Ⅱ期/Ⅲ期临床试验亦在进一步探索其临床应用价值。本文就当前非小细胞肺癌免疫疗法原理、临床试验、不良反应及待解决问题作一概述。

## Abstract (en)

Globally, Lung cancer is the leading cause of cancer-related death of high morbidity and mortality with poor prognosis, which needs some more effective and less toxic therapies. The immunotherapies offer a novel approach for the treatment of patients with non-small cell lung cancer (NSCLC) in both the adjuvant and palliative disease settings. A number of promising immunotherapies based on different mechanism have now been evaluated showing an increasing response rate. Moreover, further phase Ⅱ/Ⅲ clinical trials will be indicated to explore its value. These include checkpoint inhibitors (anti-CTLA4 antibody, anti-PD-1 antibody, anti-PD-L1 antibody), active vaccination (L-BLP25 liposome vaccine, Belagenpumatucel-L vaccine, MAGE-A3 protein vaccine) and adoptive vaccination (CIK cells). The purpose of this paper will draw a summary on the theory, clinical trials, toxicity and problems to be solved of the immunotherapies in NSCLC.

肺癌是全球范围内首个癌症相关性死亡因素，非小细胞肺癌（non-small cell lung cancer, NSCLC）约占肺癌总数80%-85%，吸烟、环境污染（厨房油烟、煤炭燃烧、汽车尾气）等构成肺癌发病的主要原因。早期接受治疗NSCLC患者超过40%会出现肿瘤复发，因此晚期NSCLC患者5年生存率不到15%，预后较差^[1]^。以铂类为基础的标准化疗方案治疗NSCLC，患者肿瘤缓解率仅为20%-35%，中位生存期（median overall survival, mOS）为10个月-12个月，分子靶向治疗与化疗方案相比，可延长NSCLC患者无疾病生存期（progression-free survival, PFS），但患者mOS未获益^[2]^。因此标准治疗或因严重不良反应（恶心、呕吐、骨髓毒性等）或因经济学毒性（分子靶向药物价格较昂贵）已使其处于治疗瓶颈水平，急需开发一种新的NSCLC疗法。目前免疫治疗NSCLC在Ⅰ期/Ⅱ期临床试验中结果良好：肿瘤缓解率提高、毒副作用小、患者易耐受，这将为NSCLC治疗开发新领域。

## 1 抗CTLA4抗体

细胞毒T淋巴细胞抗原4（cytotoxic T lymphocyte-associated antigen-4, CTLA4）是由*CTLA4*基因编码的一种跨膜蛋白质，其配体为B7-1（CD80）及B7-2（CD86）分子蛋白，可降低T细胞活性、阻碍T细胞活化通道发挥肿瘤免疫抑制作用^[3]^。2011年抗CTLA4抗体（Ipilimumab）被美国食品与药品监督管理局作为首个治疗恶性黑色素瘤免疫药物批准上市。

近年来，抗CTLA4抗体治疗NSCLC Ⅱ期/Ⅲ期临床试验迅速开展，如Lynch等^[4]^报道了一项随机双盲多中心Ⅱ期临床试验，入组204例Ⅲ期/Ⅳ期NSCLC患者（未接受化疗），按1:1:1随机分为3组，诱导期同步治疗组静脉给予Ipilimumab+PC方案化疗4周期，后续以安慰剂+PC方案化疗2周期，序贯治疗组静脉给予安慰剂+PC化疗2周期，后续以Ipilimumab+PC方案化疗4周期，对照组静脉给予安慰剂+PC化疗6周期，1次/3周，18周后予Ipilimumab或安慰剂维持治疗，直至病情进展或患者出现不可耐受性不良反应，旨在评价Ipilimumab联合PC方案治疗NSCLC的疗效。该研究发现：序贯治疗组较对照组免疫相关性无疾病生存期（immune-related PFS, irPFS）有统计学差异（5.7个月 *vs* 4.6个月，HR=0.72，*P*=0.05），但序贯治疗组较同步治疗组irPFS无统计学差异（5.7个月 *vs* 5.5个月，HR=0.81，*P*=0.13）。序贯治疗组、同步治疗组、对照组PFS分别为5.1个月、4.1个月、4.2个月，3级/4级不良反应发生率分别为15%、20%、6%。因此研究者认为：序贯Ipilimumab+PC方案可提高患者irPFS、PFS。鉴于此，Reck等^[5]^开展了一项随机双盲多中心Ⅲ期临床试验，试验计划入组920例Ⅳ期/复发肺鳞癌患者，试验组给予抗CTLA4抗体（Ipilimumab）联合PC方案化疗6周期，对照组等量给予生理盐水联合PC方案化疗6周期，后续以Ipilimumab、生理盐水维持，直至病情进展。旨在研究一线Ipilimumab联合PC方案是否较单纯PC方案使患者生存获益，试验结果值得期待。此外，单药抗CTLA4抗体试验亦在探索，如一项随机开放Ⅱ期临床试验（NCT01471197），针对晚期NSCLC（除鳞癌）患者，旨在探索一线应用抗CTLA4抗体是否较培美曲赛使患者生存获益。总体来说，近年来有关抗CTLA4抗体试验多趋向于探索药物联合作用机制、药物代谢、生物预后因子如绝对淋巴细胞计数、肿瘤浸润T淋巴细胞（tumor infiltrating lymphocyte, TIL）等，这将不仅有助于开发应用抗CTLA4抗体，而且有望促进该药物实现个体化治疗^[6]^。

## 2 抗PD-1抗体

程序性死亡1（programmed death 1, PD-1）表面受体蛋白由*PDCD1*基因编码，包括：细胞外IgV结构域、跨膜结构区域以及细胞内尾部结构[^3]^，作用于PD-1与配体PD-L1（B7-H1）、PD-L2（B7-DC）结合位点，活化CTL细胞杀伤肿瘤^[7]^。

紧随抗CTLA4抗体上市，抗PD-1抗体于2011年10月进入临床试验，已见报道有：药物安全性、不良反应、生存获益研究等。Brahmer等^[8]^报告一项剂量爬坡Ⅰ期临床试验（NCT00730639），入组127例复治NSCLC患者，分别给予抗PD-1抗体（Nivolumab）（1 mg/kg-10 mg/kg，1次/2周）12周期，旨在探讨抗Nivolumab治疗肺鳞癌组、非肺鳞癌组NSCLC的安全性及有效性，研究发现：肺鳞癌组、非肺鳞癌组患者mOS分别为9.2个月 *vs* 9.6个月，1年生存率分别为44% *vs* 41%，2年生存率分别为41% *vs* 17%，药物最佳剂量为3 mg/kg。不良反应：食欲下降（9%）、贫血（8%）、腹泻、呕吐及皮疹（7%），3级/4级不良反应：乏力、肺炎、谷草转氨酶升高（2%）。因此研究者认为：抗PD-1抗体安全性良好，复治晚期NSCLC患者OS获益明显。Gettinger等^[9]^报告了另一项随机开放Ⅲ期临床试验（NCT01673867），该试验计划入组574例一线治疗失败NSCLC（非鳞癌）患者，按1:1随机分组，一组给予抗PD-1抗体（3 mg/kg，1/12周），另一组给予多西他赛，直至病情进展或患者出现不可耐受毒副反应，旨在比较两种二线治疗方案OS获益大小。试验预计将于2015年11月完成，抗PD-1抗体试验将更着重于探索最佳适用人群、适用时机等。

## 3 抗PD-L1抗体

程序性死亡1分子配体（programmed death ligand, PD-L1）包括：PD-L1（B7-H1）、PD-L2（B7-DC），作用于PD-L1（B7-H1）与B7-1（CD80）、PD-1分子蛋白结合位点，该位点功能障碍可诱发CTL的表达，从而杀伤肿瘤细胞^[3]^。

抗PD-L1抗体治疗NSCLC临床试验大多数处于Ⅰ期/Ⅱ期，意在探索其最佳剂量、不良反应。Spigel等^[10]^报告了一项多中心剂量爬坡Ⅰ期临床试验（NCT01375842），入组53例局部晚期/转移性NSCLC患者（既往已接受手术或放疗），分别给予不同剂量抗PD-1抗体药物（1 mg/kg、3 mg/kg、10 mg/kg、20 mg/kg，1次/3周），直至1年，旨在研究单药抗PD-L1抗体（MPDL3280A）治疗NSCLC的安全性、临床活性及生物标志物。研究发现：患者中位治疗时间为106天（范围1天-324天），客观缓解率（objective response rate, ORR）为24%，24周PFS为48%，MPDL3280A疗效与PD-L1分子表达存在相关性，3级/4级不良反应发生率34%（包括心包转移、脱水、呼吸困难、乏力，肺炎、腹泻除外）。此外，MPDL3280A治疗部分NSCLC患者存在延迟性疗效。因此抗PD-L1抗体试验正在力图寻找最佳获益人群生物标志物，另外，各免疫药物之间是否存在协同抗肿瘤作用也值得进一步探索^[11]^。

## 4 L-BLP25

L-BLP25（biomira liposomal peptide 25，又称Stimuvax）包含：MUC1串联重复区域分子蛋白、非特异性辅助单磷脂酸A以及脂质体，其中异常糖基化MUC1蛋白能在癌细胞内产生新的碳水化合物表位，参与APC摄取抗原肽，促进肿瘤免疫^[12]^。

Ohyanagi等^[13]^报告了一项开放Ⅰ期/Ⅱ期研究，该研究旨在探索L-BLP25治疗Ⅲ期不可手术放化疗后NSCLC患者的安全性，研究发现：L-BLP25 1级相关不良反应为肌痛、关节痛、恶心，无严重不良反应，安全性研究与高加索裔人一致。Butts等^[14]^报告了一项随机开放多中心IIb期临床试验，研究发现：亚组接受L-BLP25+最佳支持治疗Ⅲb期NSCLC患者获益明显（mOS 30.6个月，3年存活率49%）。Wu等^[15]^报告了一项针对东亚人种随机对照双盲多中心Ⅲ期临床试验INSPIRE，试验入组Ⅲ期不可切除NSCLC患者，按2:1随机分组，试验组给予L-BLP25（930 µg，1次/周）+最佳支持治疗，对照组给予安慰剂（930 µg，1次/周）+最佳支持治疗，共8次治疗，后续以6次维持治疗直至病情进展，旨在研究试验组是否较对照组提高患者生存。相关数据将有待进一步公布。此外，Mitchel等^[16]^报告了另一项随机双盲Ⅲ期临床试验START：该试验入组1, 513例Ⅲ期不可切除性NSCLC患者（既往已接受化放疗病情未进展），按2:1随机分为2组，试验组给予L-BLP25（806 µg，1次/周），对照组以同样方式给予安慰剂处理，直至病情进展，旨在研究试验组是否较对照组患者OS获益。研究发现：试验组于对照组中位PFS（9.6个月 *vs* 7.7个月，95%CI：0.755-0.990，*P*=0.036）、至治疗失败时间（time to treatment failure, TTF）有统计学差异（试验组：HR=0.844，95%CI：0.715-0.966，*P*=0.045；对照组：HR=0.977，95%CI：0.784-1.217，*P*=0.835）。因此研究者认为：虽然试验未达预期目标，但两组PFS、TTF差异提示同步放化疗较序贯放化疗患者对L-BLP获益明显。因此下一步试验应着重研究同步放化疗患者免疫治疗影响因素。

## 5 Belagenpumatucel-L

Belagenpumatucel-L（Lucanix）是一种混合型肺癌细胞株疫苗，经带有转化生长因子β2（transforming growth factor-β2, TGF-β2）的反义核苷酸基因质粒转染，能够降低细胞内TGF-β2浓度、增加自然杀伤细胞及树突状细胞的免疫活性，发挥机体对肿瘤细胞的免疫杀伤作用^[11]^。

基于药物安全性、有效性研究之上，Giaccone等^[17]^报告了一项随机对照双盲多中心Ⅲ期临床试验，该试验入组532例Ⅲa期/Ⅲb期、Ⅳ期NSCLC患者（已接受一线含铂药物化疗无进展，距末次化疗时间4周-17.4周不等），按1:1随机分组，分别给予Lucanix、安慰剂，直至病情进展或出现不可耐受性不良反应。该研究发现：两组mOS未见明显统计学差异（药物组 *vs* 安慰剂组：20.3个月 *vs* 17.8个月，*P*=0.594），*Cox*回归分析提示：距末次化疗时间影响患者生存（*P*=0.002），肿瘤分期、放疗与否、组织学类型等亦是预后因素。因此研究者认为：试验主要预期目标虽未完成，但肺鳞癌、大细胞肺癌患者OS有统计学意义，这将确立下一步试验研究方向。

## 6 MAGE-A3

恶性黑色素瘤A3（melanoma associated antigen-A3, MAGE-A3）是一种纯化重组蛋白质疫苗，表达于35%NSCLC肿瘤细胞表面，包含MAGE-A3全蛋白^[11]^及免疫佐剂AS02_B_。

Vansteenkiste等^[18]^报告了一项随机双盲安慰剂对照Ⅱ期临床试验，旨在评价MAGE-A3蛋白质疫苗治疗可手术切除NSCLC患者的临床疗效、免疫反应及安全性。试验共入组182例术后MAGE-A3阳性表达Ib期/Ⅱ期NSCLC患者，按2:1随机分2组，试验组（*n*=122）肌注MAGE-A3蛋白质疫苗+免疫佐剂，共13次/ > 27个月，对照组（*n*=60）以同样方式给予安慰剂。研究发现：试验组、对照组中位术后44个月肿瘤复发率分别为35%、43%，两组无疾病干预期无明显统计学差异（HR=0.75，95%CI：0.46-1.23，双尾*P*=0.254）；两组OS亦没有统计学差异（HR=0.81, 95%CI: 0.47-1.40, *P*=0.454）。70个月中位随访期结果类似。MAGE-A3蛋白质疫苗副反应不明显。因此研究者认为：此小样本试验提示术后给予MAGE-A3蛋白质疫苗不良反应小，疗效肯定。一项随机双盲Ⅲ期临床试验MAGRIT（NCT00480025），计划入组2, 270例Ib期/Ⅱ期/Ⅲa期NSCLC肿瘤切除患者，旨在探索MAGE-A3疫苗能否使NSCLC肿瘤切除患者治疗获益，目前相关数据尚未发表。此外，Ulloa-Montoya等^[19]^报告了基因标志物GS，可预测MAGE-A3蛋白质疫苗疗效，该研究发现：在GS阳性表达NSCLC患者中，MAGE-A3疫苗联合免疫佐剂AS02B治疗术后NSCLC患者较安慰剂组疗效有统计学差异（HR=0.42, 95%CI: 0.36-1.97, *P*=0.70）。基因标志物将是下一步研究方向，预示免疫治疗个体化探索时代的到来。

## 7 CIK细胞

细胞因子诱导的杀伤（cytokine-induced killer, CIK）细胞治疗可在体内加强机体主动免疫、体外大量增殖并直接参与肿瘤杀伤过程，目前在我国发展较为成熟。

目前针对CIK细胞免疫治疗NSCLC的临床试验较多，设计方案不再局限探索药物安全性、有效性，更多地倾向研究药物给予方式（如药物量、频次、单药或联合用药的方式及时机等），如：Yang等^[20]^报告了一项单队列研究，入组122例Ⅲ期/Ⅳ期NSCLC患者，试验组给予NP方案化疗4周期，后续以DC+CIK细胞治疗（1次/2月），对照组仅给予NP化疗方案4周期，旨在评价体外DC诱导CIK细胞联合NP方案的有效性。研究发现：试验组较对照组明显增加CD3^+^CD56^+^细胞数量、增强机体抗肿瘤作用；此外，试验组较对照组1年生存率、2年生存率有统计学差异（57.2% *vs* 37.7%, *P*＜0.05; 27.0% *vs* 10.1%, *P*＜0.05），因此研究者认为：DC诱导CIK细胞联合NP化疗使晚期NSCLC患者临床获益。Zhong等^[21]^报告了另一项病例对照研究，试验入组60例Ⅲb期-Ⅳ期NSCLC患者，试验组给予NP方案化疗4周期，后续以自体DC/CIK细胞（> 2次/月），对照组给予NP方案化疗4周期，后续以自体DC/CIK细胞（2次/月），旨在探讨试验组较对照组TTP、OS是否存在统计学差异。研究发现：试验组较对照组获益明显（1年生存率63.3% *vs* 56.7%，2年生存率30.0% *vs* 13.3%，3年生存率23.3% *vs* 6.7%，*P*=0.037；TTP 7.3个月 *vs* 6.2个月，*P*=0.034），药物不良反应小、患者可耐受。一项随机对照开放Ⅱ期临床试验（NCT01871480），计划入组复治50例肺腺癌患者，旨在评价CIK细胞联合吉非替尼治疗对晚期、复发、转移肺腺癌患者的有效性。

## 8 展望

免疫药物疗法作为一种新型NSCLC治疗方案（除化疗、放疗、分子靶向治疗等外），可提高肿瘤缓解率、毒副作用小、经济毒性适中、可门诊条件下给药等优点。以此类药物为基础的联合化疗或单药治疗既适用于老年器官功能差、体质较差、坚决拒绝放化疗患者，又可作为已接受多线治疗患者的最后姑息治疗，因此临床应用免疫药物将为治疗NSCLC另辟蹊径。展望未来，需要解决的问题包括：免疫药物何时启用、疗程长短、单独使用抑或联合适用、鉴别最佳获益人群、药物耐药机制及克服耐药等。
